# Comparing distance metrics for rotation using the *k*-nearest neighbors algorithm for entropy estimation

**DOI:** 10.1002/jcc.23504

**Published:** 2013-12-05

**Authors:** David J Huggins

**Affiliations:** a Theory of Condensed Matter Group, University of Cambridge, Cavendish Laboratory19 J J Thomson Avenue, Cambridge, CB3 0HE, United Kingdom; b Cambridge Molecular Therapeutics Programme, University of Cambridge, Hutchison/MRC Research CentreHills Road, Cambridge, CB2 0XZ, United Kingdom; c Department of Chemistry, University of CambridgeLensfield Road, Cambridge, UK CB2 1EW, United Kingdom

**Keywords:** statistical mechanics, entropy, solvation, *k*-nearest neighbors, distance metric, molecular dynamics

## Abstract

Distance metrics facilitate a number of methods for statistical analysis. For statistical mechanical applications, it is useful to be able to compute the distance between two different orientations of a molecule. However, a number of distance metrics for rotation have been employed, and in this study, we consider different distance metrics and their utility in entropy estimation using the *k*-nearest neighbors (KNN) algorithm. This approach shows a number of advantages over entropy estimation using a histogram method, and the different approaches are assessed using uniform randomly generated data, biased randomly generated data, and data from a molecular dynamics (MD) simulation of bulk water. The results identify quaternion metrics as superior to a metric based on the Euler angles. However, it is demonstrated that samples from MD simulation must be independent for effective use of the KNN algorithm and this finding impacts any application to time series data.

## Introduction

Metrics for defining the distance between sample points are an important concept for statistical analysis and have utility in numerous algorithms. In the context of statistical mechanics, one can consider a metric describing the distance between two poses of a molecule. However, although the distance between two points in Euclidean space is well-understood and is simple to calculate from basic trigonometry, the distance between two orientations is more complicated. Inhomogeneous fluid solvation theory (IFST) is a statistical mechanical method for calculating solvation free energies by quantifying the effect of a solute acting as a perturbation to bulk solvent.^1^,^2^ The solvent is commonly water and IFST has proven useful in understanding hydration phenomena,^3^,^4^ explaining binding affinity,^5^,^6^ and calculating hydration free energies.^7^,^8^ The solvation entropy is calculated in terms of translational and orientational ordering of solvent molecules in the solute reference frame (solute-water terms) and translational and orientational ordering of solvent molecules relative to one another (water-water terms). In this work, we study the solute-water orientational entropy and do not consider the other three entropy terms commonly calculated by IFST: the solute-water translational entropy, water-water translational entropy, and water-water orientational entropy.

In IFST, the solute-water orientational entropy has generally been estimated by integrating correlation functions using a histogram method. However, histogram methods suffer from two fundamental and related problems. The first problem is that the widths of the histogram bins must be sufficient to capture the underlying probability density function (PDF). Bins that are too large are unable to describe sharply peaked PDFs and will underestimate the entropy. Conversely, bins that are too small require vast amounts of sampling to reach convergence and will otherwise overestimate the entropy.^3^,^9^ This inherent bias is the second problem with the histogram method. Recent work has highlighted these problem in relation to estimation of the solute-water orientational entropy using IFST.^8^ One alternative to this histogram method is to estimate the entropy using the *k*-nearest neighbors (KNN) algorithm.^10^,^11^ KNN provides an asymptotically unbiased estimate of the entropy and can deal effectively with sharply peaked PDFs.^12^–^14^ The KNN algorithm is suitable for entropy estimation in numerous contexts and has found applications in genetics,^15^ stenography,^16^ and astronomy.^17^ KNN has also been identified as superior to a histogram method in the context of IFST.^4^,^18^ However, the KNN algorithm estimates the probability density at a sample point by calculating the shortest distance to any other sample point and, thus, requires a distance metric to be defined. In this study, we consider a number of distance metrics for rotations in three-dimensional (3D) space and their suitability for application in the KNN algorithm. Each distance metric is compared with the histogram method for three datasets; uniform randomly generated data, biased randomly generated data, and data from a molecular dynamics (MD) simulation of bulk water.

## Methods

In this article, we consider two methods for estimating the entropy of a set of sample points, where each sample point is a rotation in 3D space. The first involves binning the sample points in a uniform histogram and the second involves estimating the density at sample points by considering a series of nearest neighbors. In the following work, the term absolute entropy refers to the Shannon entropy and the term relative entropy refers to the entropy relative to a uniform distribution.

### Entropy estimation from histogram sampling

Using a histogram method, the relative orientational entropy (H^histogram^) can be calculated by numerical integration using the Euler angles (*ω*).

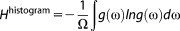
(1)

The orientational correlation functions *g*(*ω*) can be calculated by computing *α*, cos*β*, and *γ* in the laboratory reference frame for each sample point. *Ω* is the integral over the Euler angles. The limits of integration for a rotation are [0, 2π] for *α*, [−1, 1] for cos*β*, and [0, 2π] for *γ*. We used an angular bin size of 45°, leading to 8, 4, and 8 angular bins for *α*, cos*β*, and *γ* and, thus, 256 angular bins in total. Histogramming is the most commonly applied method in the context of IFST.^6^,^7^,^19^

### Entropy estimation from KNN

The KNN algorithm provides an unbiased estimate of the absolute entropy from the general expression in eq. (2).^10^

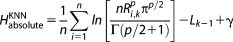
(2)

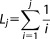
(3)*n* is the number of samples, *R*_i,k_ is the distance between sample point *i* and its *k*-th nearest neighbor, *p* is the number of degrees of freedom (three in this case), Γ is the gamma function, *L*_0_ is 0, and *γ* is Euler’s constant. Γ(5/2) is equal to 3/4π^1/2^. To compute the relative entropy (H^KNN^), eq. (2) must be corrected by the total angular volume Ω = 8π^2^.^4^,^20^

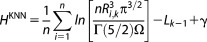
(4)

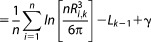
(5)

A key aspect of the KNN algorithm is the definition of a distance metric. A number of distance metrics in 3D rotational space are considered in section “Metrics for 3D rotation” below. The nearest neighbor distances were calculated by an exhaustive search of all distances at all sample points.

## Metrics for 3D Rotations

A position in 3D Euclidean space can be defined as a vector relative to the origin. The distance between two points (**a** and **b**) in 3D Euclidean space is calculated using the Euclidean metric in eq. (6):

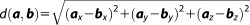
(6)

An orientation in 3D Euclidean space can be defined as a rotation relative to a reference orientation. In this work, we use a reference orientation with the primary axis aligned with the *z* axis and the secondary axis aligned with the *y* axis. We consider three representations of a rotation. The Euler angles, the quaternion representation, and the matrix representation. The unit quaternion representation (*w*, *x*, *y*, *z*) of a rotation of *θ*° about a unit vector axis (*i*, *j*, *k*) is given by 7.^21^

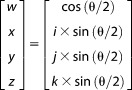
(7)

The matrix representation of this rotation is given by (8).

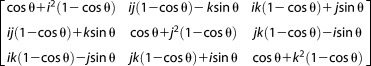
(8)

Orientations can also be defined using spherical coordinates, Hopf coordinates, or axis and angle representations.^22^ The distance between two orientations (**1** and **2**) can be derived by calculating the distance between the two rotations that bring them to the same reference orientation. However, rotations are described by Riemannian geometry rather than Euclidean geometry and a number of alternatives for calculating the distance between two rotations have been used previously. A number of these metrics are considered by Huynh.^23^ In this article, we consider four of these distance metrics and their utility for the KNN algorithm.

### Euclidean distance between the Euler angles

If Rotation 1 is described by the Euler angles **α**_1_**, β**_1_**, γ**_1_ and Rotation 2 is described by the Euler angles **α**_2_**, β**_2_**, γ**_2_**,** then the Euclidean difference (Δ_1_) can be defined as:

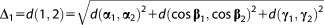
(9)

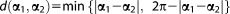
(10)

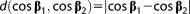
(11)

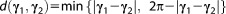
(12)

|**x**| represents the absolute value of the variable **x**. To avoid the problems of ambiguous representation, **α** and **γ** are in the range [0, 2π] and **β** is in the range [0, π]. Δ_1_ takes the range of values {0, √(4 + 2π^2^)}. A Euclidean distance metric has been used previously for KNN entropy estimation in the context of IFST.^4^

### Norm of the difference of quaternions

This metric (Δ_2_) defines the distance between two rotations as twice the Euclidean distance between the two unit quaternion representations (**q**_1_ and **q**_2_) of the rotations.^24^

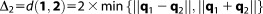
(13)

||**q**|| represents the Euclidean norm of the quaternion **q**. The minimum operator is required because the unit quaternions **q** and **−q** represent the same rotation. Δ_2_ takes the range of values {0, 2√2}.

### Geodesic on the unit sphere

This metric (Δ_3_) employs the matrix representations of the two rotations (**R**_1_ and **R**_2_) and is the natural Riemannian metric for the rotation group.^25^

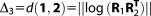
(14)

||**M**|| represents the Euclidean (Frobenius) norm of the matrix **M** and **M**^T^ represents the transpose of the matrix **M**. As shown by Huynh, Δ_3_ can be calculated more simply from the shortest arc between the two rotations on the S^3^ hypersphere using the inverse cosine of the inner product of the two unit quaternion representations (**q**_1_ and **q**_2_) of the rotations.^23^

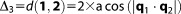
(15)

Δ_3_ takes the range of values {0, π} and is twice the value of the metric used by Wunsch.^26^

### Deviation from the identity matrix

This metric (Δ_4_) also employs the matrix representations of the two rotations (**R**_1_ and **R**_2_).^27^

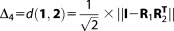
(16)

**I** represents the identity matrix. Δ_4_ takes the range of values {0, 2}.

## Efficiency Considerations

The efficiency of using metrics Δ_1_, Δ_2_, and Δ_3_ can be increased significantly by performing the square root or cosine functions only on the nearest neighbor to each sample point rather than on all distances. In the case of Δ_3_, this means identifying the largest value of the absolute inner product.

## Randomly Generated Test Data

The methods were first assessed using randomly generated data. Random orientations were created from a random axis and angle, which were generated from three random numbers between 0 and 1 (r_1_, r_2_, r_3_) using eqs. (17)–(19).

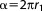
(17)

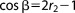
(18)

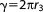
(19)

These were then used to generate a random unit vector (*x*,*y*,*z*) for the principal axis using eqs. (20)–(22).

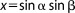
(20)

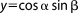
(21)

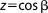
(22)

The rotation around the principal axis was determined by the *γ* angle. To generate biased data with known entropy, *r*_2_ can be divided by a divisor A.

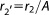
(23)

The relative entropy of the resulting PDF can be calculated using eq. (24).

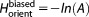
(24)

This expression is derived from an A-fold increase in probability density within 1/A of the sample space. The probability density is zero in the remainder of the space. Each test was performed 1000 times to calculate a mean and standard deviation for the relative entropy estimate. The data can also be biased by restricting the rotations to a specified distance from the reference orientation. This requires defining the distance metric and here we have used the natural metric of the shortest arc between the two rotations on the S^3^ hypersphere. This is the geodesic distance. The maximum distance was specified by the divisor B.

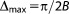
(25)

In this case, the orientations were generated using eqs. (17)–(19) and orientations with a distance greater than Δ_max_ were discarded. The entropy of the resulting set of orientations can be calculated from the remaining fraction of the total PDF. The uniform PDF for rotations can be derived from the uniform PDF for quaternions which is equal to the integrated hypersurface probability density on the S^3^ hypersphere.^28^ The area of half the S^3^ hypersphere (A_total_) is π^2^ and the area of the two caps of the S^3^ hypersphere with solid angle *φ* relative to a pole (A_caps_) is given by eq. (26).^28^,^29^ The area for two caps is required because the group S^3^ is a double cover for the rotation group SO(3) and the two caps with solid angle φ represent the same set of rotations. The following derivations are based on Ref.^28^.

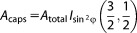
(26)

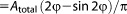
(27)

*I* is the regularized incomplete beta function.

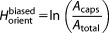
(28)

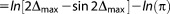
(29)

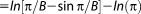
(30)

Each test was performed 1000 times to calculate a mean and standard deviation for the entropy estimate.

## MD Simulation

The methods were also assessed using data from MD simulations of bulk water. A water molecule in bulk should have no preferred orientation in the laboratory reference frame and thus the orientational distribution in the laboratory reference frame should be random and the contribution of the solute-water relative orientational entropy should be zero. We use the TIP4P-2005 water model.^30^

### System setup

The first stage was to generate a unit cell of bulk water. To generate a reasonable initial water density, a water shell of radius 50.0 Å was generated around the origin with the SOLVATE program version 1.0 (http://www.mpibpc.mpg.de/grubmueller/solvate) from the Max Planck Institute. The resulting water globule was then cut to a rhombic dodecahedral unit cell with side lengths of 25.0 Å containing 364 water molecules. To standardize the geometries of the water molecules, every hydrogen atom was deleted and all the necessary hydrogen atoms and lone pairs were built using the appropriate geometry for TIP4P-2005 water. No ions were included in the systems.

### Equilibration

Equilibration was performed for 1.0 ns in an NPT ensemble at 300 K and 1 atm using Langevin temperature control and Nosé–Hoover^31^ Langevin piston pressure control.^32^ The system was brought to equilibrium before continuing, by verifying that the energy fluctuations were stable. MD simulations were performed using an MD time step of 2.0 fs. Electrostatic interactions were modeled with a uniform dielectric and a dielectric constant of 1.0 throughout the equilibration and production runs. Van der Waals interactions were truncated at 11.0 Å with switching from 9.0 Å. Electrostatics were modeled using the particle mesh Ewald method,^33^ and the system was treated using rhombic dodecahedral periodic boundary conditions.

### Simulation

Production simulation (100.0 ns) in an NPT ensemble were performed at 300 K and 1 atm. System snapshots were saved every 10.0 fs, yielding 10,000,000 snapshots in total. MD simulations were performed using NAMD^34^ version 2.8 compiled for use with CUDA-accelerated GPUs.

### Entropy estimation

The relative orientational entropy was calculated using the histogram method and the KNN method (with each distance metric) in each of 1000 cubic voxels in a 10 × 10 × 10 Cartesian grid centred at the origin with a grid resolution was 0.5 Å.

## Rotations of Water

There are a number of additional considerations when applying these methods to calculate the contribution of solute-water correlations to the thermodynamic entropy of water. The first involves accounting for symmetry and the second involves converting the Shannon entropies to Gibbs entropies.

### The effect of symmetry

Due to the *C*_2v_ symmetry of the water molecule, rotations by *γ* angles less than π are equivalent to rotations by *γ* + π. For the histogram method, the limits of integration are reduced to [0, π] for *γ*. For an angular bin size of 45°, this leads to 8, 4, and 4 angular bins for *α*, cos*β*, and *γ* and, thus, 128 angular bins in total. For the KNN method using metric Δ_1_, Ω = 4π^2^ and eqs. (31) and (32) must be used.

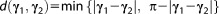
(31)

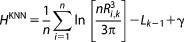
(32)

For the quaternion-based metrics (Δ_2_, Δ_3_, and Δ_4_), rotations by *θ* angles less than π are equivalent to rotations by *θ* + π and *θ* is replaced by 2*θ* in eq. (7). In terms of the randomly generated biased data, only one half of the random number affects the data and the divisor must thus be doubled in eq. (23).

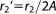
(33)

### Conversion to molar entropy

IFST provides a means to calculate the contribution of the solute-water orientational entropies to the molar solvation free energy in a given subvolume. This can be calculated using the relative orientational entropy (*H*_orient_) calculated using the histogram method or the KNN method.

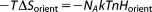
(34)

*T* is the temperature (298 K), *N*_A_ is Avogadro’s number, *k* is Boltzmann’s constant, and *n* is the mean number of water molecules within the subvolume, derived from the MD simulation. The orientational correlation functions are assumed to be independent of the position within the subvolume. It is important to note that higher-order correlations must be included to calculate the total orientational entropy of water. The higher-order relative entropy terms (such as the water-water relative orientational entropy that is typically calculated by IFST) are not zero in bulk water.

## Results and Discussion

The histogram method is compared with the KNN algorithm using four distance metrics by considering randomly generated data and data from MD simulations of bulk water. The results for randomly generated data in this section are for randomly generated water data and, thus, include the measures described in section “System setup.” The results for normal rotations are given in Supporting Information and show almost identical behavior.

### The effect of increasing K

**Figure 1:**
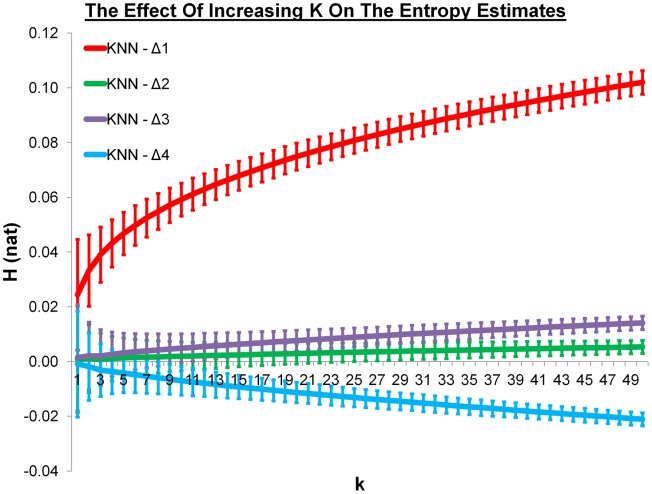
The KNN entropy estimates between *k* = 1 and *k* = 50 for 6400 randomly generated data points using the four distance metrics. Δ_1_ is in blue, Δ_2_ is in red, Δ_3_ is in green, and Δ_4_ is in purple. The entropy has natural units and the error bars represent one standard deviation from 1000 repeats of the process. [Color figure can be viewed in the online issue, which is available at wileyonlinelibrary.com.]

It is well-known for the KNN algorithm that increasing *k* will increase the precision but decrease the accuracy. We explored this by considering a range of *k* values between 1 and 50 for a fixed number (6400) of randomly generated rotations. The results for the four different metrics can be seen in Figure 1. As expected, the estimate at *k* = 1 is the closest to the expected relative entropy of zero for all four metrics but the standard deviation decreases as *k* increases. It is notable that metric 1 is significantly farther from zero than the other three metrics for all *k* values. It is also interesting that the entropies are always negative for the Δ_4_ metric and always positive for the other three metrics.

### The effect of increasing sampling

**Figure 2:**
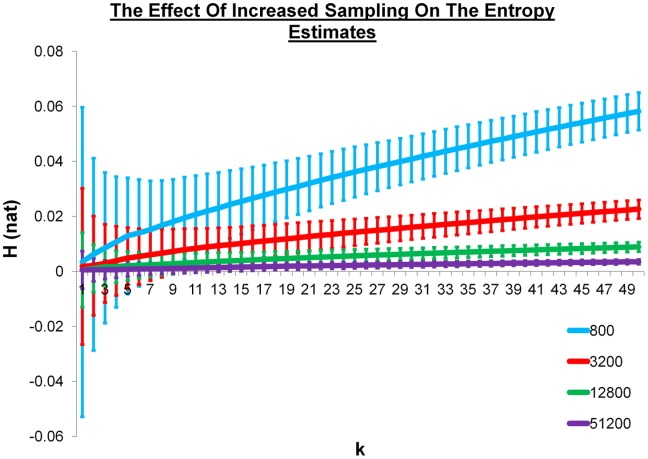
The KNN entropy estimates between *k* = 1 and *k* = 50 for 800 (cyan), 3200 (red), 12,800 (green), and 51,200 (purple) randomly generated samples using the distance metric Δ_3_. The entropy has natural units and the error bars represent one standard deviation from 1000 repeats of the process. [Color figure can be viewed in the online issue, which is available at wileyonlinelibrary.com.]

The next step was to consider the effect of increasing the amount of sampling for a given metric. We explored this by considering a range of *k* values between 1 and 50 for 800, 3200, 12,800, and 51,200 randomly generated rotations. The results for Δ_3_ can be seen in Figure 2. The expected relative entropy is again zero. As expected, the estimates improve and the standard deviations decrease as the amount of sampling increases. Again, the estimate at *k* = 1 is the closest to the expected value of zero for all four levels of sampling. As we are looking for alternatives to a histogram method and bias is one of the main problems we wish to avoid, we will only consider the *k* = 1 estimates from this point forth.

### Comparison of the metrics for uniform random data

**Figure 3:**
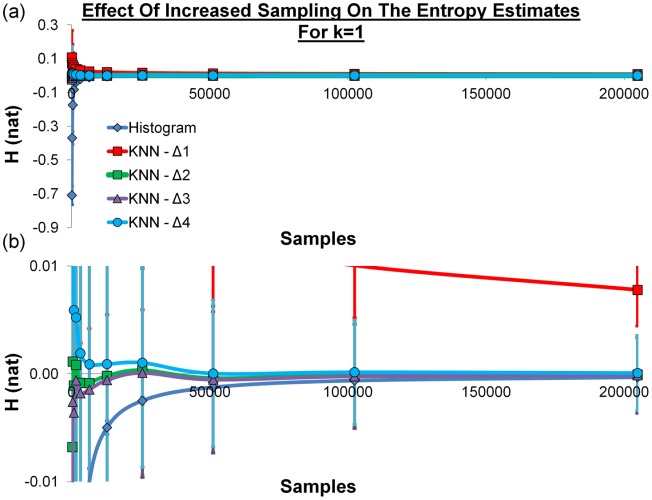
a) The histogram and KNN entropy estimates with *k* = 1 for 100, 200, 400, 800, 1600, 3200, 6400, 12,800, 25,600, 51,200, 102,400, and 204,800 randomly generated data points using the four distance metrics. The histogram estimates are represented as a blue line and diamonds, the Δ_1_ estimates are represented as a red line and squares, the Δ_2_ estimates are represented as a green line and squares, the Δ_3_ estimates are represented as a purple line and triangles, and the Δ_4_ estimates are represented as a cyan line and circles. The entropy has natural units and the error bars represent one standard deviation from 1000 repeats of the process and (b) the data for H between the limits of −0.01 and 0.01.

We first wished to compare the *k* = 1 KNN relative entropy estimates against a histogram method using randomly generated data. Figure 3 shows the comparison for different numbers of sample points. The results show that using metric Δ_1_ leads to significantly poorer performance. Metrics Δ_2_ and Δ_3_ appear to oscillate around zero and metric Δ_4_ shows a slight offset. The histogram method has a lower standard deviation than any of the KNN methods but the estimate is farther from zero for all levels of sampling for every metric except Δ_1_ and the performance suffers greatly when few data points are sampled. Metrics Δ_2_ and Δ_3_ perform well even with very few samples.

### Comparison of the metrics for biased random data

In addition to reducing bias, it is also important that the entropy estimates are accurate for nonuniform PDFs. We explored this by calculating the relative entropies for a set of rotations that were biased to certain regions of the parameter space. This was achieved using eq. (23), and the true relative entropies were calculated using eq. (24). Table 1 shows the results for the *k* = 1 KNN relative entropy estimates and the histogram method. As expected, the histogram method is unable to describe the sharply peaked PDFs and drastically underestimates the relative entropy when the divisor is large. This of course depends on the number of bins used (128 in this case) but is indicative of the problem with the histogram method. The KNN algorithm performs well with the metrics Δ_2_, Δ_3_, and Δ_4_, providing a reasonable estimate of the true relative entropy even when the divisor A is large. Conversely, the performance of the metric Δ_1_ deteriorates as the PDFs become more sharply peaked. The standard deviations are much smaller for the histogram method, as noted above for the uniform distribution, and are very similar for the four KNN metrics. This finding was explored further by biasing the orientations using eq. (25). Table 2 shows the results for the *k* = 1 KNN relative entropy estimates. Metrics Δ_2_, Δ_3_, and Δ_4_ again display the best performance, with the performance of metric Δ_1_ deteriorating slightly as the PDFs become more sharply peaked. It is important to note that the geodesic distance is used to generate the data in this case and it is thus unsurprising that metrics based on the geodesic distance perform well. However, this is the correct approach to limit a set of rotations and the conclusions are thus relevant to real data.

**Table 1:** The histogram and KNN relative entropy estimates with *k* = 1 using the four distance metrics for 25,600 randomly generated data points.

		Histogram		KNN − Δ_1_		KNN − Δ_2_		KNN − Δ_3_		KNN − Δ_4_	
A	True H	Mean	SD	Mean	SD	Mean	SD	Mean	SD	Mean	SD
1	0.00	0.00	0.00	0.02	0.01	0.00	0.01	0.00	0.01	0.00	0.01
2	−0.69	−0.69	0.00	−0.67	0.01	−0.68	0.01	−0.68	0.01	−0.68	0.01
4	−1.39	−1.39	0.00	−1.35	0.01	−1.36	0.01	−1.36	0.01	−1.36	0.01
8	−2.08	−1.39	0.00	−2.02	0.01	−2.05	0.01	−2.05	0.01	−2.05	0.01
16	−2.77	−1.39	0.00	−2.67	0.01	−2.74	0.01	−2.74	0.01	−2.74	0.01
32	−3.47	−1.39	0.00	−3.31	0.01	−3.43	0.01	−3.43	0.01	−3.43	0.01
64	−4.16	−1.39	0.00	−3.90	0.01	−4.12	0.01	−4.12	0.01	−4.12	0.01
128	−4.85	−1.39	0.00	−4.38	0.01	−4.81	0.01	−4.81	0.01	−4.81	0.01
256	−5.55	−1.39	0.00	−4.66	0.01	−5.50	0.01	−5.50	0.01	−5.50	0.01
512	−6.24	−1.39	0.00	−4.78	0.01	−6.18	0.01	−6.18	0.01	−6.18	0.01
1024	−6.93	−1.39	0.00	−4.83	0.01	−6.87	0.01	−6.87	0.01	−6.87	0.01
2048	−7.62	−1.39	0.00	−4.84	0.01	−7.55	0.01	−7.55	0.01	−7.55	0.01
4096	−8.32	−1.39	0.00	−4.85	0.01	−8.24	0.01	−8.24	0.01	−8.24	0.01
8192	−9.01	−1.39	0.00	−4.85	0.01	−8.92	0.01	−8.92	0.01	−8.92	0.01
16384	−9.70	−1.39	0.00	−4.85	0.01	−9.60	0.01	−9.60	0.01	−9.60	0.01
32768	−10.40	−1.39	0.00	−4.85	0.01	−10.28	0.01	−10.28	0.01	−10.28	0.01

The range of r_2_ was restricted using eq. (23) with the value of A reported in the table. The true relative entropies were calculated using eq. (24). The process was repeated 1000 times to calculate a mean and SD which are reported in the table for each case. The relative entropies have natural units.

**Table 2:** The KNN relative entropy estimates with *k* = 1 using the four distance metrics for 25,600 randomly generated data points.

		KNN − Δ_1_		KNN − Δ_2_		KNN − Δ_3_		KNN − Δ_4_	
B	True H	Mean	SD	Mean	SD	Mean	SD	Mean	SD
1	0.000	0.018	0.010	0.000	0.010	0.000	0.010	−0.001	0.010
2	−1.705	−1.653	0.010	−1.685	0.009	−1.685	0.009	−1.685	0.009
3	−2.853	−2.791	0.010	−2.832	0.010	−2.832	0.010	−2.832	0.010
4	−3.692	−3.620	0.010	−3.671	0.010	−3.671	0.010	−3.671	0.010
5	−4.350	−4.269	0.010	−4.328	0.010	−4.328	0.010	−4.328	0.010
6	−4.891	−4.801	0.010	−4.869	0.010	−4.869	0.010	−4.869	0.010
7	−5.350	−5.249	0.010	−5.328	0.009	−5.328	0.009	−5.328	0.009
8	−5.748	−5.637	0.010	−5.726	0.010	−5.726	0.010	−5.726	0.010
9	−6.100	−5.979	0.010	−6.078	0.010	−6.078	0.010	−6.078	0.010
10	−6.415	−6.283	0.010	−6.393	0.009	−6.393	0.009	−6.393	0.009
11	−6.700	−6.557	0.010	−6.678	0.010	−6.678	0.010	−6.678	0.010
12	−6.960	−6.807	0.010	−6.938	0.010	−6.938	0.010	−6.938	0.010
13	−7.200	−7.035	0.010	−7.178	0.010	−7.178	0.010	−7.178	0.010
14	−7.422	−7.245	0.010	−7.400	0.010	−7.400	0.010	−7.400	0.010
15	−7.629	−7.440	0.010	−7.606	0.010	−7.606	0.010	−7.606	0.010
16	−7.822	−7.623	0.010	−7.800	0.010	−7.800	0.010	−7.800	0.010

The distributions were restricted using eq. (25) with the value of B reported in the table. The true relative entropies were calculated using eq. (30). The process was repeated 1000 times to calculate a mean and SD which are reported in the table for each case. The relative entropies have natural units.

### Comparison of the metrics for data from MD

**Figure 4:**
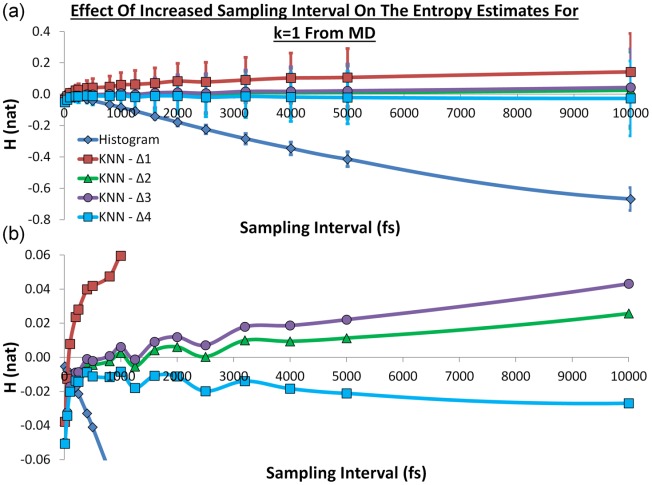
a) The histogram and KNN entropy estimates with *k* = 1 using the four distance metrics from the MD simulation. The sampling intervals were 10, 50, 100, 200, 250, 400, 500, 800, 1000, 1250, 1600, 2000, 2500, 3200, 4000, 5000, and 10,000 fs. The histogram estimates are represented as a blue line and diamonds, the Δ_1_ estimates are represented as a red line and squares, the Δ_2_ estimates are represented as a green line and squares, the Δ_3_ estimates are represented as a purple line and triangles, and the Δ_4_ estimates are represented as a cyan line and circles. The entropy has natural units and the error bars represent one standard deviation from the 1000 voxels and b) The data for H between the limits of −0.01 and 0.01.

Although the analysis of random data is very revealing, useful application of the KNN algorithm for statistical mechanics requires that it functions with data sampled from simulation. We tested this by performing a 100-ns MD simulation of bulk water and considering the solute-water orientational correlation function of water molecules in small voxels. The solute-water relative orientational entropy should be zero, as the water molecules have no preferred orientation in the laboratory reference frame. However, there is an additional concern that must be considered in this case. For the randomly generated data, each sample point is independent of every other. For the MD simulation, samples that are close together in time will be highly correlated. This will not affect the accuracy of a histogram method, as long as sufficient samples are taken. However, it will affect the KNN method because the correlation between orientations in snapshots that are close together in time will lead to closer nearest neighbours and, thus, skewed entropy estimates. This effect is independent of how many samples are taken. This issue has not been reported previously and the requirement for independent samples requires careful analysis of the sampling frequency. In our simulation, 10,000,000 samples were taken from the 100-ns simulation corresponding to a sampling interval of 10 fs. Figure 4 shows the effect of decreasing the sampling frequency on the relative entropy estimates for the histogram method and the KNN algorithm using the four metrics across 1000 voxels. The histogram methods behave as expected, with the most accurate estimate using the largest number of samples at a sampling interval of 10 fs. The histogram estimates worsen rapidly as the sampling frequency decreases. Metric Δ_1_ makes notably poorer estimates than the other three KNN distance metrics (as observed for the randomly generated data in Fig. 1) and diverges from zero as the number of samples decreases. This can be seen more clearly in Figure 4b. The performances of the other three metrics show interesting behavior and are comparable, though metrics Δ_2_ and Δ_3_ appear to be superior. As noted above, the performance is weaker at high and low sampling frequencies, leading to a peak in performance for sampling frequencies between 400 and 1250 fs. In this range, the samples are sufficiently uncorrelated to be effectively independent, but contain enough information to yield an accurate entropy estimate.

### Application to thermodynamic predictions

To really understand the utility of these methods, we explored the effect on the predicted thermodynamic properties. As discussed, the solute-water relative orientational entropy should make zero contribution to the molar free energy of bulk water. This contribution can be calculated for each voxel from the orientational entropies using eq. (33). The 1000 voxels correspond to a total volume of 125 Å^3^ and the contribution from this volume can be computed by summing the contribution of each voxel. This data can be seen in Table 3. In the window of 400–1250 fs, metrics Δ_2_ and Δ_3_ make the closest estimates to zero and are better than the histogram estimate with a 10 fs sampling interval. Although the histogram estimate with a 10 fs sampling interval of + 0.013 kcal/mol may seem a reasonable error, it is important to note that 125 Å^3^ is a small volume in the context of molecular simulation. Results suggest that solutes perturb the surrounding water to a distance of two or three solvation shells.^8^^,^^35^–^37^ Even for a very small solute, the region of interest has an approximate radius of 12 Å and the volume of interest is approximately 7250 Å^3^. Extrapolating the error, this volume would lead to an error of + 0.75 kcal/mol. This is very similar to the results found in recent studies.^8^ Because the KNN method is unbiased, the error is not expected to be extensive and increasing the volume is actually expected to decrease the total error, as the average will tend toward the mean. However, the estimate in each voxel is expected to have a large standard deviation and be less reliable than the total.

**Table 3:** The thermodynamic estimates of −*TΔS*_orient_ using the histogram method and KNN with *k* = 1 from the MD simulation for the 1000 voxels using the four distance metrics.

Sampling interval (fs)	−*TΔS*_orient_ (kcal/mol)				
	Histogram	KNN − Δ_1_	KNN − Δ_2_	KNN − Δ_3_	KNN − Δ_4_
10	0.013	0.093	0.123	0.123	0.125
50	0.018	0.031	0.081	0.079	0.084
100	0.026	−0.019	0.044	0.042	0.049
200	0.043	−0.058	0.025	0.022	0.034
250	0.052	−0.069	0.025	0.021	0.035
400	0.081	−0.098	0.007	0.002	0.022
500	0.101	−0.103	0.011	0.005	0.027
800	0.164	−0.117	0.006	−0.002	0.028
1000	0.207	−0.147	−0.006	−0.015	0.020
1250	0.262	−0.155	0.013	0.003	0.044
1600	0.346	−0.174	−0.010	−0.023	0.026
2000	0.437	−0.206	−0.015	−0.029	0.028
2500	0.548	−0.193	−0.001	−0.018	0.048
3200	0.694	−0.224	−0.025	−0.044	0.034
4000	0.844	−0.254	−0.022	−0.045	0.046
5000	1.012	−0.262	−0.027	−0.053	0.053
10,000	1.635	−0.350	−0.059	−0.102	0.069

The sampling intervals were 10, 50, 100, 200, 250, 400, 500, 800, 1000, 1250, 1600, 2000, 2500, 3200, 4000, 5000, and 10,000 fs.

## Conclusions

The KNN algorithm is a very appealing method for entropy estimation, due to being unbiased and having modest sampling requirements. In this study, we have highlighted the importance of using a suitable distance metric for the calculation and explored four distance metrics for rotation in 3D. This allowed us to explore the entropy associated with a dataset of orientations, as each orientation can be represented as a rotation from a reference orientation. The relative entropy estimates for the KNN method with the four metrics was then compared to a histogram method. The results of the study apply to all molecules with a single C_2_ primary axis (such as the C_2v_ point group of water). The results in the Supporting Information apply to molecules with no rotational axis of symmetry (such as those in the C_1_ point group) and are very similar leading to the same conclusions. It is also worth noting that the KNN method for entropy estimation is a useful test of the suitability of distance metrics, using the approach described here.

Before summarizing the findings, it is worth examining the assumptions. A major assumption is that the random orientations are actually random. In truth, random number generation is not entirely random and thus the relative entropy is not zero. However, the results suggest that the relative entropy is very close to zero and thus estimates far from zero are inaccurate. One of the main findings of the study is that the distance metric based on Euler angles (Δ_1_) is not as effective as distance metrics based on quaternions (Δ_2_, Δ_3_, and Δ_4_). Tables 1 and 2 show that the three quaternion metrics yield identical results (to two decimal places). Thus, metrics Δ_2_ and Δ_4_ yield very similar distances to the natural metric Δ_3_. Good distance metrics will yield the same distance as the natural distance metric for sufficiently close points. This suggests that metrics Δ_2_ and Δ_4_ will be good distance metrics, given sufficient sampling such that the NN distances are small. Conversely, distance metrics based on Euler angles are flawed because the summed difference between the individual angle components of two rotations can be large in cases where the two rotations are very similar.^23^,^38^ This is true both for uniform and sharply peaked PDFs. The ability to model uniform PDFs is important in the statistical mechanical modeling of solvent regions far from the solute and the ability to model sharply peaked PDFs is important in the statistical mechanical modeling of solvent regions near complex solutes. Further studies in this area should consider how sharply peaked the orientational PDFs of water are in the complex environment surrounding a solute or protein binding site. This will highlight the effectiveness of the distance metrics. It is worth noting that the relative entropy for metric Δ_1_ also converges to zero in Figure 3 and this suggests that Δ_1_is a good distance metric but requires significantly more sampling than the quaternion metrics to reach the same level of accuracy. Further work should also consider alternatives to the KNN algorithm, or extensions to it such as kernel-density estimation.^39^,^40^

Another finding, which is not unexpected, is that for the randomly generated data the *k* = 1 estimates are closer to zero than estimates with larger *k* values but have larger standard deviations. Real-world applications of KNN need to consider the balance of accuracy and precision that is desired. As is also expected, increased sampling of the randomly generated data improves the performance of the KNN algorithm for any value of *k*. However, when considering the data from MD simulations, the results highlight the necessity for the samples to be independent. This finding is relevant to KNN entropy estimation in any context. For the simulation of bulk TIP4P-2005 water at 298 K and 1 atm, sampling intervals less than 400 fs yields correlated samples and impairs performance even though more samples are taken. A key advantage of the KNN algorithm is the lack of bias. Although the bias in the histogram method leads to an extensive error as the volume of the system increases, the performance of the KNN algorithm is expected to improve with increased volume. However, for each voxel the KNN entropy estimate may show significant deviation due to the high variance and this may affect the utility of visualising the contributions of different regions to solvation free energies.

In summary, the results of this study identify the quaternion metrics as superior to the metric based on the Euler angles, for solute-water orientational entropy estimation. These results are applicable to any entropy calculations that consider orientational correlations and are also of interest for torsional correlations. Importantly, sufficient samples of independent data must be taken to achieve optimal performance of the KNN algorithm with time series data.
