# Supplementary material for: Comparing distance metrics for rotation using the k-nearest neighbors algorithm for entropy estimation
Source: J Comput Chem. 2013 Dec 5;35(5):377–85. doi: 10.1002/jcc.23504 (PMC4238811; doi:10.1002/jcc.23504)
Supplement: Supplementary file 1 [file jcc0035-0377-sd1.docx]

Supplementary Information For "Comparing Distance Metrics For Rotation Using The k-Nearest Neighbours Algorithm For Entropy Estimation"


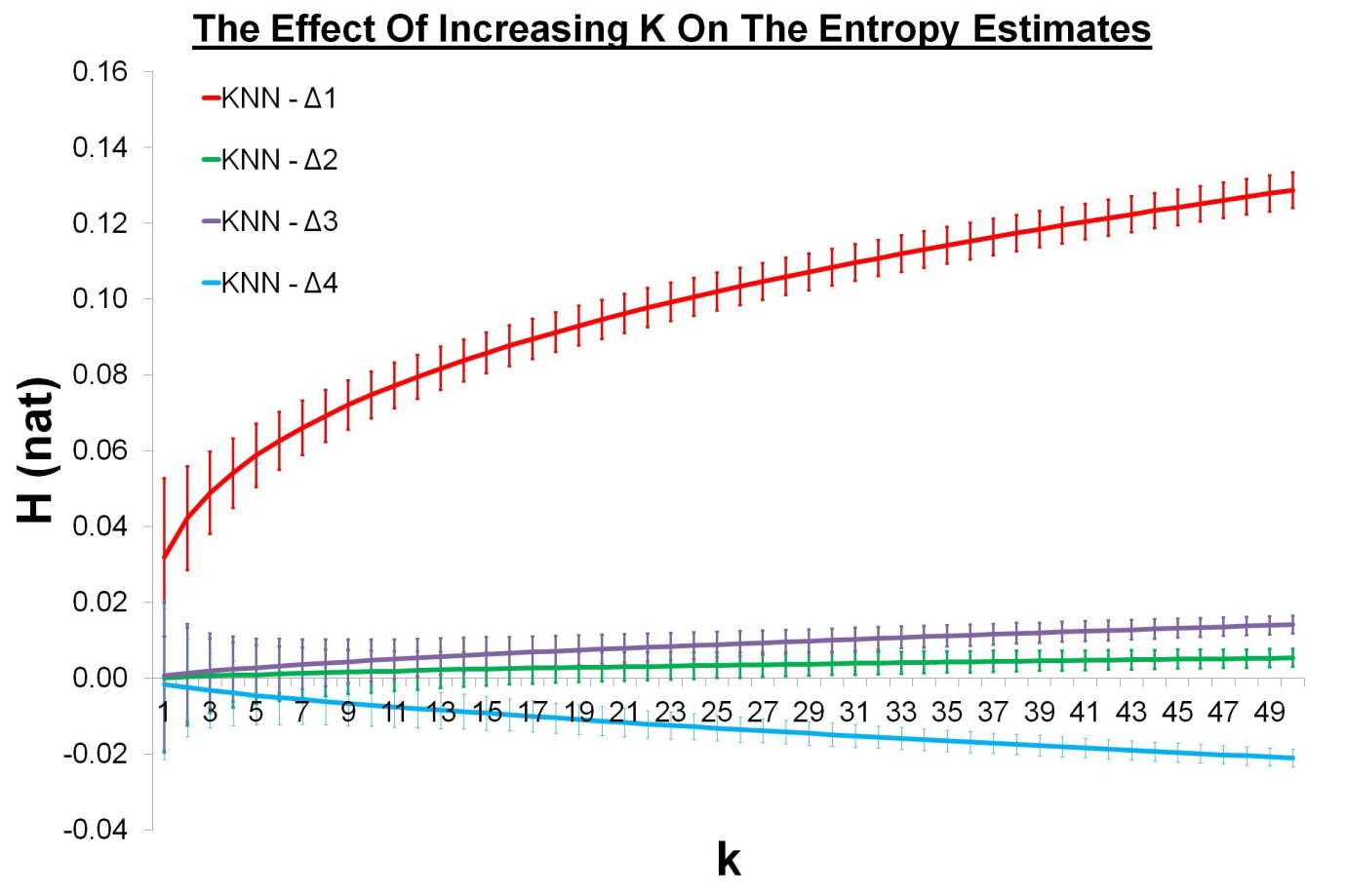
Figure S1 – The KNN relative entropy estimates between k=1 and k=50 for 6400 randomly generated data points using the four distance metrics. Δ_1_ is in blue, Δ_2_ is in red, Δ_3_ is in green, and Δ_4_ is in purple. The relative entropy has natural units and the error bars represent one standard deviation from 1000 repeats of the process.


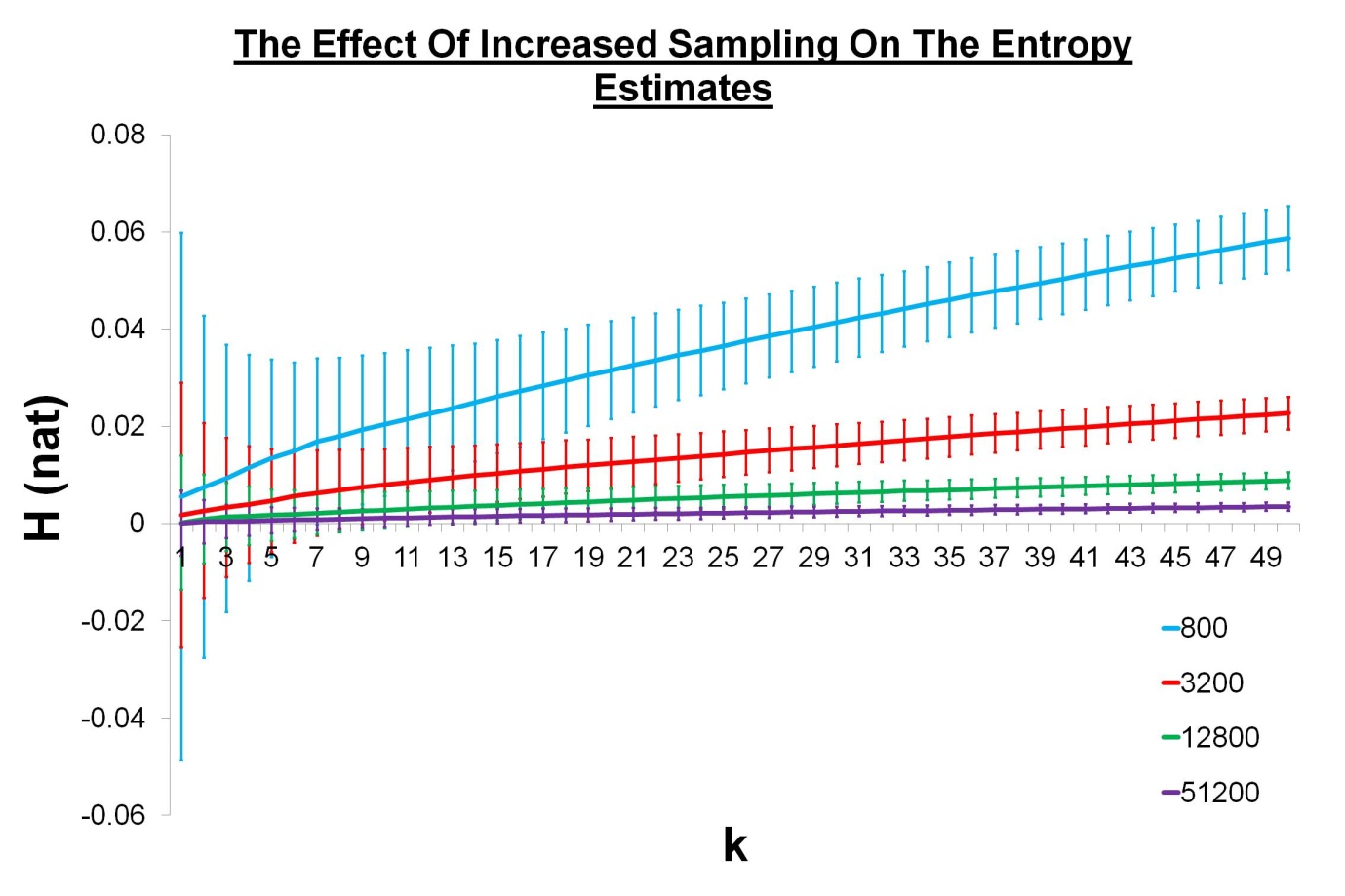
**Figure S2 – The KNN relative entropy estimates between k=1 and k=50 for 800 (cyan), 3200 (red), 12800 (green), and 51200 (purple) randomly generated samples using the distance metric Δ_3_. The relative entropy has natural units and the error bars represent one standard deviation from 1000 repeats of the process.**


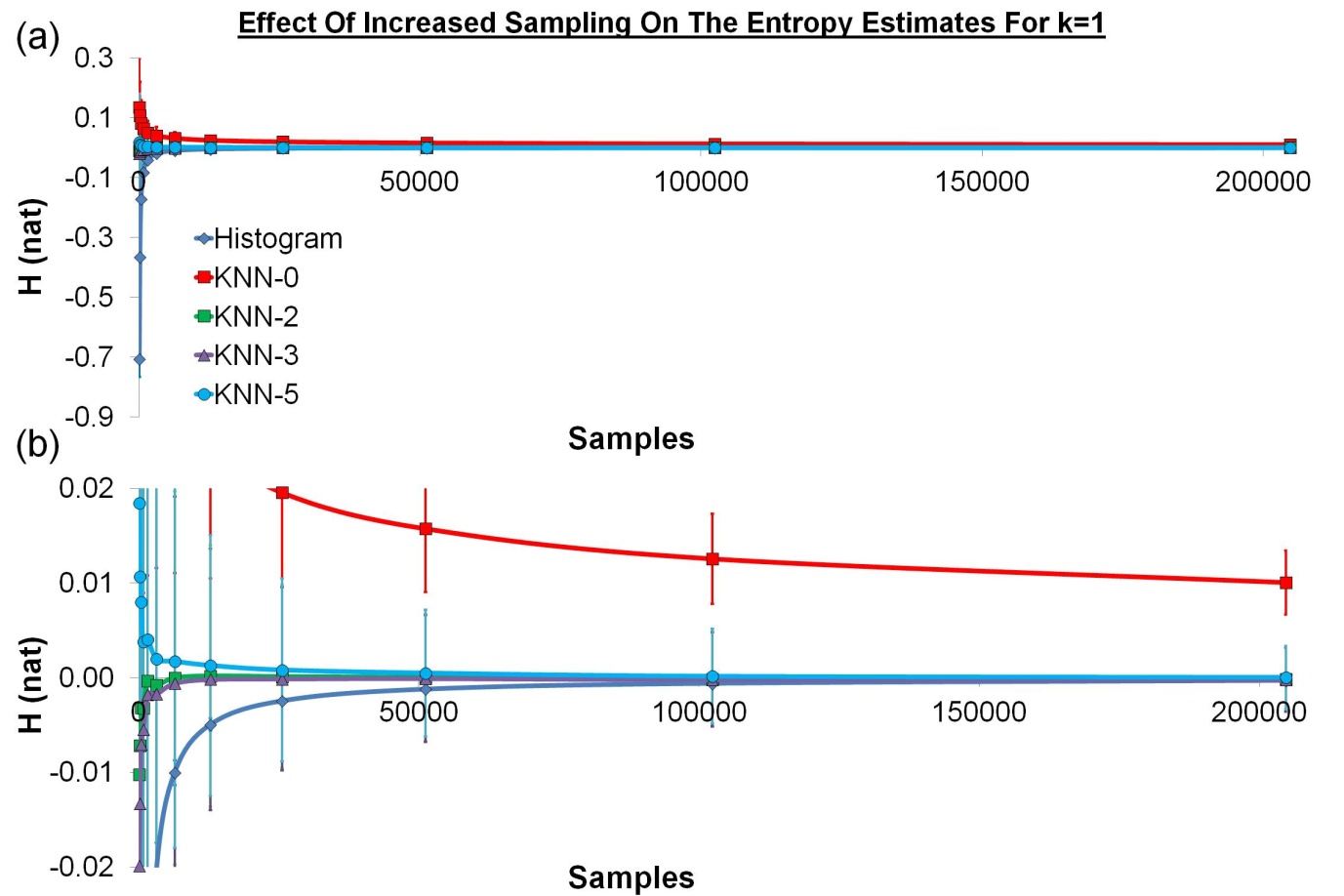
**Figure S3 – (a) The histogram and KNN relative entropy estimates with k=1 for 100, 200, 400, 800, 1600, 3200, 6400, 12800, 25600, 51200, 102400, and 204800 randomly generated data points using the four distance metrics. The histogram estimates are represented as a blue line and diamonds, the Δ_1_ estimates are represented as a red line and squares, the Δ_2_ estimates are represented as a green line and squares, the Δ_3_ estimates are represented as a purple line and triangles, and the Δ_4_ estimates are represented as a cyan line and circles. The relative entropy has natural units and the error bars represent one standard deviation from 1000 repeats of the process. (b) The data for H between the limits of -0.02 and 0.02.**

| **A** | **True H** | **Histogram** | | **KNN - Δ1** | | **KNN - Δ2** | | **KNN - Δ3** | | **KNN - Δ4** | |
| --- | --- | --- | --- | --- | --- | --- | --- | --- | --- | --- | --- |
|  |  | *Mean* | *SD* | *Mean* | *SD* | *Mean* | *SD* | *Mean* | *SD* | *Mean* | *SD* |
| 1 | 0.00 | 0.00 | 0.00 | 0.02 | 0.01 | 0.00 | 0.01 | 0.00 | 0.01 | 0.00 | 0.01 |
| 2 | -0.69 | -0.69 | 0.00 | -0.66 | 0.01 | -0.68 | 0.01 | -0.68 | 0.01 | -0.68 | 0.01 |
| 4 | -1.39 | -1.39 | 0.00 | -1.34 | 0.01 | -1.36 | 0.01 | -1.36 | 0.01 | -1.37 | 0.01 |
| 8 | -2.08 | -1.39 | 0.00 | -2.00 | 0.01 | -2.05 | 0.01 | -2.05 | 0.01 | -2.05 | 0.01 |
| 16 | -2.77 | -1.39 | 0.00 | -2.65 | 0.01 | -2.74 | 0.01 | -2.74 | 0.01 | -2.74 | 0.01 |
| 32 | -3.47 | -1.39 | 0.00 | -3.27 | 0.01 | -3.43 | 0.01 | -3.43 | 0.01 | -3.43 | 0.01 |
| 64 | -4.16 | -1.39 | 0.00 | -3.82 | 0.01 | -4.12 | 0.01 | -4.12 | 0.01 | -4.12 | 0.01 |
| 128 | -4.85 | -1.39 | 0.00 | -4.20 | 0.01 | -4.81 | 0.01 | -4.81 | 0.01 | -4.81 | 0.01 |
| 256 | -5.55 | -1.39 | 0.00 | -4.39 | 0.01 | -5.49 | 0.01 | -5.49 | 0.01 | -5.49 | 0.01 |
| 512 | -6.24 | -1.39 | 0.00 | -4.46 | 0.01 | -6.18 | 0.01 | -6.18 | 0.01 | -6.18 | 0.01 |
| 1024 | -6.93 | -1.39 | 0.00 | -4.49 | 0.01 | -6.87 | 0.01 | -6.87 | 0.01 | -6.87 | 0.01 |
| 2048 | -7.62 | -1.39 | 0.00 | -4.50 | 0.01 | -7.55 | 0.01 | -7.55 | 0.01 | -7.55 | 0.01 |
| 4096 | -8.32 | -1.39 | 0.00 | -4.50 | 0.01 | -8.24 | 0.01 | -8.24 | 0.01 | -8.24 | 0.01 |
| 8192 | -9.01 | -1.39 | 0.00 | -4.50 | 0.01 | -8.92 | 0.01 | -8.92 | 0.01 | -8.92 | 0.01 |
| 16384 | -9.70 | -1.39 | 0.00 | -4.50 | 0.01 | -9.60 | 0.01 | -9.60 | 0.01 | -9.60 | 0.01 |
| 32768 | -10.40 | -1.39 | 0.00 | -4.50 | 0.01 | -10.28 | 0.01 | -10.28 | 0.01 | -10.28 | 0.01 |

Table S1 – The histogram and KNN relative entropy estimates with k=1 using the four distance metrics for 25600 randomly generated data points. The range of r_1_ was restricted using equation 23 with the value of A reported in the table. The true relative entropies were calculated using equation 24. The process was repeated 1000 times to calculate a mean and standard deviation (SD) which are reported in the table for each case. The relative entropies have natural units.

| B | True H | KNN - Δ1 | | KNN - Δ2 | | KNN - Δ3 | | KNN - Δ4 | |
| --- | --- | --- | --- | --- | --- | --- | --- | --- | --- |
|  |  | Mean | SD | Mean | SD | Mean | SD | Mean | SD |
| 1 | 0.000 | 0.019 | 0.010 | 0.000 | 0.010 | 0.000 | 0.010 | -0.001 | 0.010 |
| 2 | -1.705 | -1.653 | 0.010 | -1.686 | 0.010 | -1.686 | 0.010 | -1.686 | 0.010 |
| 3 | -2.853 | -2.789 | 0.009 | -2.832 | 0.010 | -2.832 | 0.010 | -2.832 | 0.010 |
| 4 | -3.692 | -3.615 | 0.010 | -3.670 | 0.010 | -3.670 | 0.010 | -3.670 | 0.010 |
| 5 | -4.350 | -4.261 | 0.010 | -4.329 | 0.010 | -4.329 | 0.010 | -4.329 | 0.010 |
| 6 | -4.891 | -4.789 | 0.010 | -4.869 | 0.010 | -4.869 | 0.010 | -4.869 | 0.010 |
| 7 | -5.350 | -5.234 | 0.010 | -5.328 | 0.010 | -5.328 | 0.010 | -5.328 | 0.010 |
| 8 | -5.748 | -5.619 | 0.010 | -5.726 | 0.010 | -5.726 | 0.010 | -5.726 | 0.010 |
| 9 | -6.100 | -5.957 | 0.010 | -6.078 | 0.010 | -6.078 | 0.010 | -6.078 | 0.010 |
| 10 | -6.415 | -6.257 | 0.010 | -6.393 | 0.010 | -6.393 | 0.010 | -6.393 | 0.010 |
| 11 | -6.700 | -6.528 | 0.011 | -6.678 | 0.010 | -6.678 | 0.010 | -6.678 | 0.010 |
| 12 | -6.960 | -6.774 | 0.011 | -6.938 | 0.010 | -6.938 | 0.010 | -6.938 | 0.010 |
| 13 | -7.200 | -6.998 | 0.011 | -7.178 | 0.010 | -7.178 | 0.010 | -7.178 | 0.010 |
| 14 | -7.422 | -7.205 | 0.010 | -7.400 | 0.009 | -7.400 | 0.009 | -7.400 | 0.009 |
| 15 | -7.629 | -7.397 | 0.010 | -7.607 | 0.010 | -7.607 | 0.010 | -7.607 | 0.010 |
| 16 | -7.822 | -7.574 | 0.010 | -7.800 | 0.010 | -7.800 | 0.010 | -7.800 | 0.010 |

Table S2 – The KNN relative entropy estimates with k=1 using the four distance metrics for 25600 randomly generated data points. The distributions were restricted using equation 25 with the value of B reported in the table. The true relative entropies were calculated using equation 30. The process was repeated 1000 times to calculate a mean and standard deviation (SD) which are reported in the table for each case. The relative entropies have natural units.
